# Privacy-preserving AUC computation in distributed machine learning with PHT-meDIC

**DOI:** 10.1371/journal.pdig.0000753

**Published:** 2025-11-13

**Authors:** Marius de Arruda Botelho, Cem Ata Baykara, Ali Burak Ünal, Nico Pfeifer, Mete Akgün

**Affiliations:** 1 Methods in Medical Informatics, Department of Computer Science, University of Tübingen, Tübingen, Germany; 2 Medical Data Privacy and Privacy-Preserving ML on Healthcare Data, Department of Computer Science, University of Tübingen, Tübingen, Germany; 3 Institute for Translational Bioinformatics, University Hospital Tübingen, Tübingen, Germany; Weill Cornell Medicine, UNITED STATES OF AMERICA

## Abstract

Ensuring privacy in distributed machine learning while computing the Area Under the Curve (AUC) is a significant challenge because pooling sensitive test data is often not allowed. Although cryptographic methods can address some of these concerns, they may compromise either scalability or accuracy. In this paper, we present two privacy-preserving solutions for secure AUC computation across multiple institutions: (1) an exact global AUC method that handles ties in prediction scores and scales linearly with the number of samples, and (2) an approximation method that substantially reduces runtime while maintaining acceptable accuracy. Our protocols leverage a combination of homomorphic encryption (modified Paillier), symmetric and asymmetric cryptography, and randomized encoding to preserve the confidentiality of true labels and model predictions. We integrate these methods into the Personal Health Train (PHT)-meDIC platform, a distributed machine learning environment designed for healthcare, to demonstrate their correctness and feasibility. Results using both real-world and synthetic datasets confirm the accuracy of our approach: the exact method computes the true AUC without revealing private inputs, and the approximation provides a balanced trade-off between computational efficiency and precision. All relevant code and data is publicly available at https://github.com/PHT-meDIC/PP-AUC, facilitating straightforward adoption and further development within broader distributed learning ecosystems.

## Author summary

A commonly used metric to evaluate the performance of machine learning models is the Area Under the Curve (AUC). Calculating the AUC in distributed machine learning settings is challenging because data cannot be shared between institutions due to privacy concerns. To address this, we developed two privacy-preserving methods: one that calculates the exact AUC securely and another that provides faster approximations with high accuracy. These methods use advanced encryption techniques to protect sensitive data while enabling secure collaboration. We tested them in a real-world healthcare platform called PHT-meDIC and demonstrated their effectiveness. The code and data is publicly available at https://github.com/PHT-meDIC/PP-AUC to support wider adoption.

## 1 Introduction

The Area Under the Curve (AUC) is one of the most widely used metrics for evaluating the performance of binary classifiers, providing a robust summary of a model’s ability to distinguish between positive and negative classes. In centralized machine learning settings, computing AUC is straightforward as both prediction scores and true labels are readily accessible. However, in distributed machine learning frameworks, such as federated learning [1–3], ensuring the privacy of participants’ data, particularly true labels, becomes a critical challenge. Labels often contain privacy-sensitive information, and sharing them across multiple parties in a distributed environment could lead to privacy breaches.

Most existing approaches to compute the AUC in distributed settings privacy-preserving rely on differential privacy (DP) mechanisms [4–6], which introduce noise to protect sensitive information but yield approximate AUC values. Other methods, such as ROC-GLM [7] in DataSHIELD [8], use statistical techniques but carry overhead and remain constrained to specific software ecosystems. Cryptographic solutions based on multi-party computation (MPC) [9] enable exact AUC calculation but frequently suffer from scalability issues or significant communication overheads.

This paper presents PP-AUC, two novel cryptographic methods for distributed, privacy-preserving exact (DPPE) and approximation (DPPA) AUC computation that addresses these limitations. Our exact method combines cloud-based Paillier homomorphic encryption with symmetric and asymmetric cryptography and randomized encoding to enable AUC’s secure and exact computation in distributed environments without revealing sensitive data. The approximation method utilizes similar technologies but only approximates the AUC based on a predefined number of specific thresholds used to calculate the AUC.

Unlike existing methods, DPPE-AUC can compute the global AUC across any number of participants and handle tie conditions, where multiple samples may have identical prediction scores while ensuring both accuracy and scalability. DPPA-AUC improves computational efficiency by using additively homomorphic (Paillier-based) secure aggregation to approximate the AUC, introducing only a small deviation from the exact value

We integrate DPPE- and DPPA-AUC methods within the PHT-meDIC platform [10], a distributed learning platform tailored for privacy-preserving analysis of healthcare data. To evaluate the performance of both methods, we conduct experiments on real-world healthcare datasets and synthetic data, demonstrating that our protocol achieves exact AUC computation while scaling linearly with the number of samples. All runs of the DPPE-method are compared in terms of runtime and correctness to the DPPA-AUC method. Our implementation is publicly available in Python and can be easily adapted to other distributed machine-learning systems. In the PHT-meDIC setting, the analogy of stations and trains represents data stations acting as secure endpoints where algorithms (trains) travel to access and process data locally, ensuring privacy while enabling collaborative computations.

## 2 Background

### 2.1 Area under curve

The Area Under the Receiver Operating Characteristic Curve (AUROC, or simply AUC) is a widely used metric for evaluating binary classifiers. It provides a single scalar value that summarizes the model’s ability to distinguish between positive and negative classes, independent of a specific classification threshold.

The ROC curve plots the False Positive Rate (FPR) on the x-axis and the True Positive Rate (TPR) on the y-axis. For a given threshold *i*, these statistics are defined as:


$$\text{TPR}[i]=\frac{TP[i]}{TP[i]+FN[i]},\;\text{FPR}[i]=\frac{FP[i]}{FP[i]+TN[i]}$$

where *TP*[*i*], *FP*[*i*], *FN*[*i*], and *TN*[*i*] are the numbers of true positives, false positives, false negatives, and true negatives at threshold *i*. The AUC provides a single value between 0 and 1. A score of 1.0 indicates perfect separation between the positive and negative classes. A score of 0.5 reflects performance no better than random guessing.

To compute the AUC, we evaluate the changes in TPR and FPR at each unique threshold derived from the sorted prediction scores. Let *S* denote the number of such threshold points. Then, the AUC is computed as:

$$𝒜=\frac{1}{2}\sum_{i=1}^{S}(TPR[i]+TPR[i-1])\cdot (FPR[i]-FPR[i-1])$$ (1)

Eq 1 aggregates contributions from each adjacent pair of points on the ROC curve. To reduce the number of division operations and make this expression more suitable for computation under our security constraints, we can re-express the formula as follows:

$$𝒜=\frac{1}{2\cdot T\cdot N}\sum_{i=1}^{S}(TP[i]+TP[i-1])\cdot (FP[i]-FP[i-1])$$ (2)

Here, *TP*[*i*] and *FP*[*i*] represent the cumulative true and false positives up to threshold *i*, and *T* and *N* are the total number of positive and negative samples in the dataset, respectively.

### 2.2 Modified paillier cryptosystem

The modified Paillier system [11, 12] supports the addition of two ciphertexts and the multiplication of a ciphertext with a plaintext constant. This allows users to perform computations on ciphertexts, which results in an identical output as that performed on plaintexts. In this system, the public key is (*n*,*g*,*h* = *g*^*x*^). *n* is the product of two safe primes: *z* and *y*. The secret key is $x\in [1,\frac{n^{2}}{2}]$ and *g* that is in order of $\frac{\left(z-1)(y-1\right)}{2}$ equals to –*a^2n^* where $a\in {\mathbb{Z}}_{n^{2}}^{*}$

- **Encryption:** The message $m\in {\mathbb{Z}}_{n}$ is encrypted as follows:
  $c_{1}=g^{r}\;\mathrm{mod}\;n^{2}$ and $c_{2}=h^{r}(1+mn)\;\mathrm{mod}\;n^{2}$
  where a random r∈[1,n/4].
- **Decryption:** To recover *m*, the decryption of $\left(c_{1},c_{2}\right)$ is performed as follows:

$m=dec(\frac{(c_{2}/c_{1}^{x}-1)\;\mathrm{mod}\;n^{2}}{n})$

- **Proxy Re-encryption:** The secret key *x* is split into two shares such that $x=x_{1}\;+\;x_{2}$. In the modified Paillier system the ciphertext $\left(c_{1},c_{2}\right)$ can be partially decrypted to $\left(c_{1}^{\prime },c_{2}^{\prime }\right)$ as follows:
  $c_{1}^{\prime }=c_{1}$ and $c_{2}^{\prime }=c_{2}/c_{1}^{x_{1}}\;\mathrm{mod}\;n^{2}$
  Then, $\left(c_{1}^{\prime },c_{2}^{\prime }\right)$ can be decrypted to *m* using *x*_2_ instead of *x* with the decryption method described above.

### 2.3 Homomorphic properties

The modified Paillier cryptosystem is homomorphic with respect to both addition and scalar multiplication. This means that certain operations on encrypted data correspond directly to the same operations on the underlying plaintexts without requiring the data to be decrypted first. Such homomorphic properties are fundamental in enabling secure computations on encrypted data.

- **Addition**: Let $\left(c_{1,1},c_{1,2}\right)$ be the encryption of a message *m*_1_ and $\left(c_{2,1},c_{2,2}\right)$ be the encryption of a message *m*_2_. In the modified Paillier scheme, “adding” these ciphertexts (through the group operation in ${\mathbb{Z}}_{n^{2}}$, typically component-wise multiplication) produces a new ciphertext $\left(c_{3,1},c_{3,2}\right)$.
  $\left(c_{3,1},c_{3,2})=(c_{1,1}\cdot c_{2,1}\mathrm{mod}n^{2},\;c_{1,2}\cdot c_{2,2}\mathrm{mod}n^{2}\right)$.
  Decrypting $\left(c_{3,1},c_{3,2}\right)$ yields $m_{1}\;+\;m_{2}\;\mathrm{mod}\;n$. Hence,
  $dec(c_{3,1},c_{3,2})=m_{1}\;+\;m_{2}\;(\mathrm{mod}\;n)$. This property is crucial in scenarios where multiple values must be summed securely.
- **Scalar Multiplication**: Additionally to addition, the Paillier cryptosystem also supports the multiplication of an encrypted value by a plaintext constant. Suppose $\left(c_{1},c_{2}\right)$ encrypts a message *m*, and let *k* be a known integer in ${\mathbb{Z}}_{n}$. Then raising the ciphertext to the power *k* corresponds to multiplying the underlying plaintext by *k*:

$\left(c_{1}^{k}\mathrm{mod}n^{2},\;c_{2}^{k}\mathrm{mod}n^{2})\;⟶\;m\times k\;(\mathrm{mod}\;n\right)$

  Consequently, $dec(c_{1}^{k},c_{2}^{k})=m\times k\;(\mathrm{mod}\;n)$
  This ability to apply scalar multiplication homomorphically is useful for computing weighted sums or scaling factors while preserving the confidentiality of the underlying data.

These homomorphic properties allow us to calculate the exact AUC with partial decryption in proxy re-encryption settings—without ever exposing the underlying plaintexts.

### 2.4 Randomized encoding for multiplication

One of the privacy-enhancing techniques that we use in this study is Randomized Encoding (RE) [13, 14]. The idea of RE is to use random values to hide a function’s inputs and reveal only its output. It creates components of encoding of the desired function by using random values. Then, the output of this function can only be obtained by combining these components in a certain way. There are REs for several functions in the literature for privacy-preserving machine learning [15, 16]. Among those, we benefit from the RE for *multiplication* [17].

**Definition 1 (Modified RE for Multiplication [17])**. *Let f be a function of x*_1_ *and x*_2_ *defined over a ring* 𝖱 *such that* $f(x_{1},x_{2})=x_{1}\;\cdot \;x_{2}$. *f can be perfectly encoded by the function* $\hat{f}$ *defined as follows:*


$$\hat{f}(x_{1},x_{2},r_{1},r_{2},r_{3})=(x_{1}+r_{1},x_{2}+r_{2},r_{2}x_{1}+r_{1}x_{2}+r_{1}r_{2})$$

*where $r_{1},r_{2}$ and r*_*3*_ *are uniformly chosen random values. In order to obtain the result of $y=f(x_{1},x_{2})$, one needs to compute $c_{1}\;\cdot \;c_{2}-c_{3}$ given the encoding $\left(c_{1},c_{2},c_{3}\right)$.*

### 2.5 PHT-meDIC

PHT-meDIC [10] is a secure and interoperable platform for distributed healthcare data analysis developed within the German Medical Informatics Initiative (MII) [18]. It enables privacy-preserving analytics by bringing algorithms to the data, ensuring that patient data remains locally controlled. Key advantages include strong data protection, flexible support for custom analysis code, robust governance mechanisms, and interoperability with other national platforms.

A prominent representative of distributed analytics is the Personal Health Train (PHT), created under the GO FAIR initiative [19] to enable privacy-preserving data analysis on medical data collected from hospitals and research projects. It was further developed by the German National Chapter through standards, guidelines, and reference implementations [20]. Since 2019, different PHT ecosystems have emerged within the MII. Within MII, the PHT-meDIC is one technology for distributed analysis between data integration centers (DIC), which securely host input data. PHT-meDIC is a distributed learning platform developed for privacy-preserving healthcare data analysis within the MII. Built around an ’algorithm-to-data’ paradigm, it ensures that sensitive patient information remains under local control at each institution, eliminating the need for data pooling or replication. The platform’s container-based approach facilitates the secure execution of complex analytics while reducing infrastructure modifications at local hospital sites. Furthermore, the PHT-meDIC is interoperable [21] with other DIC distributed learning platforms such as PADME [22]. PHT-meDIC employs data encryption at rest and follows an honest but curious threat model to safeguard sensitive healthcare data. Moreover, it signs each algorithm and maintains a chain of digital signatures, ensuring no manipulation throughout the distributed analysis process. In addition, PHT-meDIC provides built-in governance mechanisms,such as mandatory code review and approval workflows, to uphold compliance and maintain trust among participating institutions. The key generation within the PHT-meDIC platform can be directly handled by the user within the desktop app for symmetric encryption protocols (RSA) and Paillier keys or, as in our case, within an initialization station. This station does not contribute data to the analysis but handles tasks like additional key creation (Cloud-Paillier key components).

### 2.6 Related work

DP has become a popular framework for protecting sensitive data in federated analysis, as it offers well-defined privacy guarantees by adding controlled noise [23]. However, DP approaches typically compute approximate metrics, leading to discrepancies between a model’s theoretical and actual performance. Besides the approximation approach, DP method [24] assumes the proxy server is honest-but-curious and local or global input can be obtained. Some recent efforts explore hybrid methods that combine DP with secure aggregation or limited cryptographic primitives to improve accuracy [25]. However, these hybrid strategies can still introduce computational and communication overhead

Statistical models like ROC-GLM [7], implemented within the DataSHIELD framework, enable distributed AUC estimation but have platform-specific dependencies and may not scale seamlessly to large datasets. In contrast, multi-party computation (MPC) protocols [9] facilitate exact computation by performing secure operations across multiple sites. Although MPC ensures that raw data remain private, it often involves complex implementation details and high communication costs. Consequently, advanced MPC systems—such as those based on homomorphic encryption—are still not widely used for large-scale AUC estimation. These constraints highlight the need for methods that maintain exactness, reduce overhead, and scale effectively across multiple sites without sacrificing privacy guarantees.

Table 1 summarizes the key characteristics of different privacy-preserving AUC computation methods, drawing upon the discussions and referenced literature within the paper. This includes the proposed DPPE-AUC and DPPA-AUC (collectively referred to as PP-AUC in the table for brevity, though their individual characteristics are detailed throughout the paper). The comparison focuses on their respective strengths, limitations, the degree of exactness they achieve in AUC computation, and the nature of the privacy guarantees they provide, offering a comparative overview against established and alternative approaches.

**Table 1:** Comparison of privacy-preserving AUC computation methods.

Method	Strengths	Limitations	Exactness	Privacy
DP (Differential Privacy) [24]	Formal privacy guarantees; lightweight.	Yields approximate AUC values; accuracy depends on noise level; assume an honest-but-curious proxy.	Approx.	DP-based guarantees
Hybrid DP [25]	Better accuracy than pure DP; added privacy layers.	introduce more computational and communication overhead; require trust in certain components.	Approx.	Moderate
ROC-GLM [7]	Simple concept; no raw data shared directly.	Platform-specific dependencies; limited scalability to large datasets; constrained to specific software ecosystems.	Approx.	Moderate (statistical disclosure control)
MPC (Multi-Party Computation) [9]	Exact AUC calculation; strong privacy guarantees as raw data remains private.	suffers from scalability issues; significant communication overheads; complex implementation details.	Exact	Strong (cryptographic)
PP-AUC (DPPE-AUC/DPPA-AUC)	DPPE: Exact AUC, handles ties, scales linearly with the number of samples. DPPA: Faster runtime, constant with respect to the number of samples for a fixed number of thresholds, with negligible accuracy deviation. Both leverage modified Paillier, symmetric/asymmetric crypto, randomized encoding. Integrated into PHT-meDIC.	Runtime increases linearly with the number of participants. DPPE: Runtime further depends on prediction value distribution and uniqueness. Security relies on no collusion between proxy and stations.	DPPE: Exact. DPPA: Approx.	Strong (cryptographic)

## 3 Materials and methods

### 3.1 Preliminaries of the protocol

Assume we have *n* participating stations (S_1_, S_2_, …, S_n_) and a initialization and proxy station S_0_ and S_P_ in the network. The initialization station S_0_ and the proxy station S_P_ do not participate in the analysis but act as an intermediary for key creation and secure computations. Each station holds its local data, and the machine learning model has already produced prediction values and randomly assigned flag subjects for privacy preservation.

Let *M* be the number of samples, and let the masked prediction values at each station be denoted as {*pre*}, where the predictions are masked as $\{pre\}=(r_{1}\;\cdot \;pre)\;+\;r_{2}$, with *r*_1_ and *r*_2_ being randomly generated values to obscure the real prediction data.

Our protocols utilize the following cryptographic functions, with specified schemes and key sizes:

- RANDS(): Generates random integers for masking and cryptographic operations.
- GEN_SYM_KEY(): Generates a 256-bit symmetric key for AES-GCM (Advanced Encryption Standard in Galois/Counter Mode).
- $ENC\_SYM(M,K_{SYM})$: Encrypts a message M using AES-GCM with the symmetric key *K*_*SYM*_.
- DEC_SYM(C,K_SYM): Decrypts a ciphertext *C* using AES-GCM with the symmetric key *K*_*SYM*_.
- $ENC\_ASYM(M,PK_{RSA})$: Encrypts a message *M* (typically a symmetric key) using RSA-OAEP (Optimal Asymmetric Encryption Padding) with a 4096-bit RSA public key *PK*_*RSA*_.
- $DEC\_ASYM(C,SK_{RSA})$: Decrypts a ciphertext *C* using RSA-OAEP with the corresponding RSA private key *SK*_*RSA*_.
- $ENC\_P(M,PK_{P})$: Encrypts a message *M* using the modified Paillier cryptosystem with a 3072-bit public key *PK*_*P*_.
- $DEC\_P(C,SK_{P})$: Decrypts a ciphertext *C* using the modified Paillier cryptosystem with the private key *SK*_*P*_.
- $ADD(C_{1},C_{2})$: A homomorphic function that performs the addition of Paillier ciphertexts *C*_1_ and *C*_2_.
- $SUB(C_{1},C_{2})$: A homomorphic function that performs the subtraction of Paillier ciphertexts *C*_1_ and *C*_2_.
- MUL(C,M): A homomorphic function that multiplies a Paillier ciphertext *C* with a plaintext *M*.

### 3.2 DPPE-AUC

**Fig 1:**
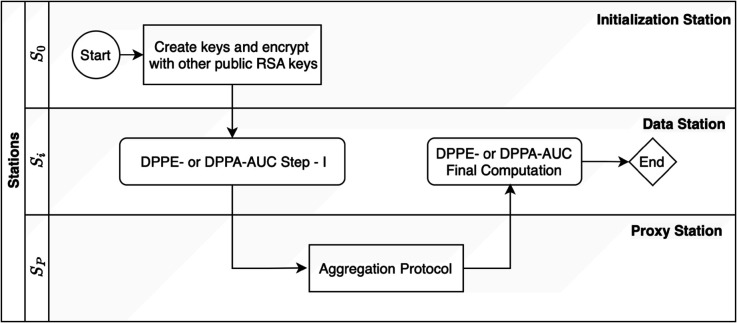
Overview of the DPPE- and DPPA-AUC Process: The workflow involves three types of stations: the initialization Station (S_0_), Data Stations (S_i_), and Proxy Station (S_Proxy_). The process starts at S_0_ with Paillier key generation and encryption using RSA public keys. S_i_ perform DPPE- or DPPA-AUC Step I, followed by an Aggregation Protocol at the S_Proxy_. The results are then processed in final computation at the S_i_, completing the protocol.

In this section, we introduce our distributed privacy-preserving method for exact AUC computation, referred to as DPPE-AUC, which has been seamlessly integrated into the PHT-meDIC platform. The workflow for both methods is shown in Fig 1.

#### Initialization.

The cryptographic setup is established once and remains persistent across multiple protocol runs. Each participating Data Station (*S*_*i*_) and the Proxy Station (*S*_*Proxy*_) generates its own long-term **RSA-4096 key pair** ($\left(PK_{S_{i}},SK_{S_{i}}\right)$ and $\left(PK_{S_{Proxy}},SK_{S_{Proxy}}\right)$, respectively) and shares its public key within the network. The Initialization Station (*S*_0_) bootstraps the homomorphic encryption system by generating a **3072-bit modified Paillier key pair** ($\left(PK_{P},SK_{P}\right)$). *S*_0_ then splits the private key *SK*_*P*_ into two shares, $SK_{P_{0}}$ and $SK_{P_{1}}$, such that $SK_{P}=SK_{P_{0}}\;+\;SK_{P_{1}}$. The public Paillier key *PK*_*P*_ is distributed to all stations. *S*_0_ securely transmits one private share ($SK_{P_{0}}$) to the Proxy Station *S*_*Proxy*_ and the other share ($SK_{P_{1}}$) to all Data Stations *S*_*i*_ by encrypting the shares with the respective stations’ public RSA keys.

This initialization process is carried out using a train (image) updated by station S_0_, which sequentially visits all stations along the route (S_1_, S_2_, …, S_n_, S_Proxy_).

#### Client computation.

During the execution of Algorithm 1, each data station *S*_*i*_ generates a new, ephemeral **256-bit AES-GCM symmetric key** (*K*_*i*_) for this specific protocol run. This per-session key is used to encrypt the station’s masked prediction values via ENC_SYM (as defined in Sect 3.1). To securely transmit this key to the Proxy, the station employs a key wrapping mechanism: it encrypts *K*_*i*_ using the proxy’s public RSA key ($PK_{S_{Proxy}}$) via ENC_ASYM. The first station generates a random value *r*_1_, used to obscure the prediction values by multiplication. At each station, an additional random component *r*_2_ is calculated as $r_{2}=(pre\;\mathrm{mod}\;r_{1})$, further masking the prediction values. These masked predictions are encrypted using the symmetric key *K*_*i*_.

**Algorithm 1: *dppe-auc* Station Protocol - Step I.**

1: **procedure** DPPE-AUC_STATION$ID_{S_{i}}$, $SK_{S_{i}}$

2:   **n** is the number of stations

3:   **m** is the number of predictions confidences

4:   **Step 1: Symmetric Key Generation**

5:   $K_{i}\leftarrow GEN\_SYM\_KEY(ID_{S_{i}})$

6:   **Step 2: Generation of Random Values**

7:   **if** *S*_*i*_ is the first station **then**

8:    $r_{1}\leftarrow RANDS()$

9:    **for** *j* = 2 to n **do**

10:     $\langle r_{1}\rangle_{j}\leftarrow ENC\_ASYM(\langle r_{1}\rangle ,PK_{S_{j}})$

11:    **end for**

12:   **else**

13:    $r_{1}\leftarrow DEC\_ASYM(\langle r_{1}\rangle_{i},SK_{S_{i}})$

14:   **end if**

15:   **Step 3: Encrypt Predictions, Labels and Flags**

16:   **for** *j* = 1 to m **do**

17:    $tmp\leftarrow r_{1}\;\cdot \;pre[j]\;+\;(pre[j]\;\mathrm{mod}\;r_{1})$

18:    $\langle pre[j]\rangle \leftarrow ENC\_SYM(tmp,K_{i})$

19:    $\langle label[j]\rangle \leftarrow ENC\_P(label[j],PK_{P})$

20:    $\langle flag[j]\rangle \leftarrow ENC\_P(flag[j],PK_{P})$

21:   **end for**

22:   **Step 4: Encrypt Symmetric Key for Proxy**

23:   $\langle K_{i}\rangle \leftarrow ENC\_ASYM(K_{i},PK_{S_{Proxy}})$

24: **end procedure**

The random value *r*_1_ is encrypted by the RSA public keys of stations other than S_0_ and S_Proxy_. Additionally, binary labels and flag values ∈{0,1} are encrypted using Paillier public key *PK*_*P*_. The encrypted results are stored within the train image and passed to the next station in the route. The subsequent stations decrypt $\langle r_{1}\rangle$ using their RSA private key. The process of obfuscating prediction values and encrypting labels and flags continues similarly across all stations, also using the same random value *r*_1_ to ensure intersite ties are computed analogously.

#### Proxy computation.

The proxy station retrieves the train image and executes Algorithm 2. First, it decrypts the symmetric keys ($\langle K_{1}\rangle$, $\langle K_{2}\rangle$, …, $\langle K_{n}\rangle$) using its private RSA key $SK_{S_{Proxy}}$. The masked prediction values from all stations are decrypted and concatenated into a single table along with the corresponding encrypted labels and flags. *S*_*P*_ then sorts this table according to the masked prediction confidences while keeping the actual prediction values hidden, ensuring that it does not access the real input data.

Based on the Paillier-encrypted labels and flag values, the proxy computes the encrypted true positive ⟨TP⟩ and false positive ⟨FP⟩ values. For each masked prediction value *pre*[*i*], the proxy sums all labels greater than *pre*[*i*] to calculate ⟨TP[i]⟩. The corresponding ⟨FP[i]⟩ value is determined by subtracting ⟨TP[i]⟩ from the sum of the flag values greater than *pre*[*i*]. This subtraction effectively eliminates the influence of dummy samples, whose labels are all set to zero and thus contribute nothing to the true positive count.

Due to the limitations of Paillier encryption, the proxy cannot directly perform multiplications on encrypted values. Instead, it delegates these computations to the stations. However, to prevent stations from inferring private information about other participants, we apply randomized encoding techniques to mask both the numerator (*N*) and denominator (*D*) values.

For the denominator *D*, the proxy computes the sum of all labels as $\langle TP_{A}\rangle$ and the sum of all flag values minus $\langle TP_{A}\rangle$ (Eq 3) as $\langle FP_{A}\rangle$. These values are multiplied by randomly generated constants *a* and *b*, respectively, using Paillier multiplication. The results are then used to compute the randomized encoding components of the denominator: $\langle D_{1}\rangle$, $\langle D_{2}\rangle$, and $\langle D_{3}\rangle$, with random integers $r_{A}^{1}$ and $r_{A}^{2}$:

$$\langle D_{1}\rangle =ADD(\langle TP_{A}\rangle ,r_{A}^{1})\;\langle D_{2}\rangle =ADD(\langle FP_{A}\rangle ,r_{A}^{2})\;\langle D_{3}\rangle =ADD(ADD(MUL(\langle TP_{A}\rangle ,r_{A}^{2}),MUL(\langle FP_{A}\rangle ,r_{A}^{1})),r_{A}^{1}\;\cdot \;r_{A}^{2})$$ (3)

For the numerator *N*, the proxy computes the difference in successive false positive values as ⟨dFP[i]⟩=⟨FP[i]⟩−⟨FP[i−1]⟩ and the sum of successive true positive values ⟨sTP[i]⟩=⟨TP[i]⟩+⟨TP[i−1]⟩ across all threshold values (Eq 4). All ⟨sTP[i]⟩ and ⟨dFP[i]⟩ values are multiplied by *a* and *b*, respectively. To handle ties in prediction values, we use the method from [9] to compute unique indices for the masked predictions. The proxy generates three random values $r_{i}^{1}$, $r_{i}^{2}$ and *z*_*i*_ for each index *i*. All *z*_*i*_ values are summed to zero. These random values are used to compute the randomized encoding components for the numerator:

$$\langle N[i{]}_{1}\rangle =ADD(\langle sTP[i]\rangle ,r_{i}^{1})\;\langle N[i{]}_{2}\rangle =ADD(\langle dFP[i]\rangle ,r_{i}^{2})\;\langle N[i{]}_{3}\rangle =ADD(ADD(MUL(\langle sTP[i]\rangle ,r_{i}^{2}),MUL(\langle dFP[i]\rangle ,r_{i}^{1})),r_{i}^{1}\;\cdot \;r_{i}^{2})$$ (4)

To improve runtime efficiency, the sum of all $\langle N[i{]}_{3}\rangle$ values is computed as $\langle N_{3}\rangle$. The proxy station then partially decrypts all random components $\langle D_{1}\rangle$, $\langle D_{2}\rangle$, $\langle D_{3}\rangle$, $\langle N[i{]}_{1}\rangle$, $\langle N[i{]}_{2}\rangle$, and $\langle N_{3}\rangle$, where i∈{1,2,…,m}, using its partial Paillier private key $SK_{P_{0}}$. These components are written to the train results, and the proxy pushes the updated image back to the stations.

**Algorithm 2: *dppe-auc* Aggregation Protocol.**

1: **procedure** DPPE-AUC_PROXY$ID_{S_{i}}$, $SK_{S_{P}}$

2:   **Step 1: Decrypt masked prediction confidences**

3:   **Step 2: Sort Paillier-encrypted labels and flags**

**according to masked prediction confidences**

4:   **Step 3: Compute True Positives (TPs) and False**

**Positives (FPs) for a unique set of masked prediction**

**values**

5:   **Step 4: Compute Denominator Components**

6:   $a,b,r_{A}^{1},r_{A}^{2}\leftarrow RANDS()$

7:   $\langle TP_{A}\rangle \leftarrow MUL(\langle TP[last]\rangle ,a)$

8:   $\langle FP_{A}\rangle \leftarrow MUL(\langle FP[last]\rangle ,b)$

9:   $\langle D_{1}\rangle \leftarrow ADD(\langle TP_{A}\rangle ,r_{A}^{1})$

10:   $\langle D_{2}\rangle \leftarrow ADD(\langle FP_{A}\rangle ,r_{A}^{2})$

11:   $\langle D_{3}\rangle \leftarrow ADD(ADD(MUL(\langle TP_{A}\rangle ,r_{A}^{2})\;+\;MUL(\langle FP_{A}\rangle ,r_{A}^{1})),r_{A}^{1}\;\cdot \;r_{A}^{2})$

12:   **Step 5: Compute Numerator Components**

13:   $\langle N_{3}\rangle \leftarrow 0$

14:   **for** *i* = 0 to m **do**

15:    $r_{i}^{1},r_{i}^{2}\leftarrow RANDS()$

16:    ⟨sTP[i]⟩←MUL(ADD(⟨TP[i]⟩,⟨TP[i−1]⟩),a)

17:    ⟨dFP[i]⟩←MUL(SUB(⟨FP[i]⟩,⟨FP[i−1]⟩),b)

18:    $\langle N[i{]}_{1}\rangle \leftarrow ADD(\langle sTP[i]\rangle ,r_{i}^{1})$

19:    $\langle N[i{]}_{2}\rangle \leftarrow ADD(\langle dFP[i]\rangle ,r_{i}^{2})$

20:    $\langle N[i{]}_{3}\rangle \leftarrow ADD(ADD(MUL(\langle sTP[i]\rangle ,r_{i}^{2})\;+\;MUL(\langle dFP[i]\rangle ,r_{i}^{1})),r_{i}^{1}\;\cdot \;r_{i}^{2}\;+\;z_{i})$

21:    $\langle N_{3}\rangle \leftarrow ADD(\langle N_{3}\rangle ,\langle N[i{]}_{3}\rangle )$

22:   **end for**

23:   **Step 6: Partially Decrypt Components**

24:   $\left(\langle N[i{]}_{1}\rangle ,\langle N[i{]}_{2}\rangle )\leftarrow DEC\_P((\langle N[i{]}_{1}\rangle ,\langle N[i{]}_{2}\rangle ),SK_{P_{0}}\right)$ i∈{1,2,…,m}

25:   $\left(\langle D_{1}\rangle ,\langle D_{2}\rangle ,\langle D_{3}\rangle ,\langle N_{3}\rangle )\leftarrow DEC\_P((\langle D_{1}\rangle ,\langle D_{2}\rangle ,\langle D_{3}\rangle ,\langle N_{3}\rangle ),SK_{P_{0}}\right)$

26: **end procedure**

#### Final computation.

Each station S_i_ pulls the updated image and extracts the partially decrypted random components and fully decrypts them using its private Paillier key $SK_{P_{1}}$. Station S_i_ can then locally compute the DPPE-AUC using the following equation:

$$\text{DPPE-AUC}=\frac{\sum_{i=1}^{M}(N[i{]}_{1}\;\cdot \;N[i{]}_{2})-N_{3}}{2\times (D_{1}\;\cdot \;D_{2}-D_{3})}$$ (5)

The randomness in the fully decrypted station values cannot be eliminated. The only way to obtain completely deterministic values is by computing the AUC using Eq 5.

### 3.3 DPPA-AUC

In this section, we provide a detailed explanation of our approximate AUC computation method, referred to as DPPA-AUC.

The use of approximate AUC for privacy-preserving model performance evaluation in distributed settings was first proposed by Sun et al. [24]. Sun et al. employed differential privacy to compute the AUC score of a global model in a federated environment. Although DPPA-AUC adopts the same underlying approximation technique, rather than differential privacy, it uses Paillier cryptosystem and randomized encoding to compute the approximate AUC value within the PHT-meDIC platform.

#### Initialization.

The initialization phase of DPPA-AUC is identical to DPPE-AUC. Each station holds an RSA key pair, and station *S*_0_ generates and splits a Paillier key pair (*PK*_*P*_, *SK*_*P*_) into $SK_{P_{0}}$ and $SK_{P_{1}}$. The public key and encrypted private key shares are distributed across the network via the same train-based mechanism.

#### Client computation.

During the execution of Algorithm 3, each station computes their TP and FP for a set of predefined decision points *D*. The number of prediction confidences is denoted as *m*.

For each decision point *D*[*i*], the algorithm accumulates the counts of *ones* (representing true labels equal to 1) and *zeros* (representing true labels equal to 0). These counts are updated while traversing the prediction values until the confidence values fall below the current decision point *D*[*i*]. If the last prediction value is reached and has not yet been visited, the algorithm ensures it is processed to finalize the counts.

The computed true positives *TP*[*i*] and false positives *FP*[*i*]) for each decision point are then encrypted using the Paillier public key *PK*_*P*_. The encrypted values ⟨TP[i]⟩ and ⟨FP[i]⟩ are passed to the next station or the proxy for further processing.

**Algorithm 3: *dppa-auc* station protocol.**

1: **procedure** DPPA-AUC_STATION$ID_{S_{i}}$, $SK_{S_{i}}$

1:   **Step 1: Compute TP and FP for Decision Points**

3:   **m** is the number of predictions confidences

4:   **D** is the set of decision points

5:   ones,zeros,p,last_one_visited←0,0,0,False

6:   **for** d **in **D **do**

7:    **while** p<mandpre[p]>d **do**

8:     ones←ones+label[p]

9:     zeros←zeros+(1−label[p])

10:     p←p+1

11:    **end while**

12:    **if** pequalsmand¬last_one_visited **then**

13:     last_one_visited←True

14:     ones←ones+label[m]

15:     zeros←zeros+(1−label[m])

16:    **end if**

17:    TP[i],FP[i]←ones,zeros

18:   **end for**

19:   **Step 2: Encrypt Data**

20:   **for** *i* = 0 to len(D) **do**

21:    $\langle TP[i]\rangle ,\langle FP[i]\rangle \leftarrow ENC\_P(TP[i],PK_{P}),ENC\_P(FP[i],PK_{P})$

22:   **end for**

23: **end procedure**

#### Proxy computation.

During the execution of Algorithm 4, the proxy station aggregates the TP and FP values across all stations for a set of decision points *D*. For each decision point *D*[*j*], the proxy computes the global sums $\langle TP_{global}[j]\rangle$ and $\langle FP_{global}[j]\rangle$ by adding the encrypted contributions $\langle TP_{S_{i}}[j]\rangle$ and $\langle FP_{S_{i}}[j]\rangle$ from all stations using the Paillier encryption scheme.

**Algorithm 4: *dppa-auc* Aggregation Protocol.**

1: **procedure** DPPA-AUC_PROXY$ID_{S_{i}}$, $SK_{S_{P}}$

2:   **D** is the set of decision points

3:   **Step 1: Aggregate TP and FP Across Stations**

4:   **for** *j* = 0 to len(D) **do**

5:    **for** each station i **do**

6:     $\langle TP_{global}[j]\rangle \leftarrow ADD(\langle TP_{global}[j]\rangle ,\langle TP_{S_{i}}[j]\rangle ,PK_{p})$

7:     $\langle FP_{global}[j]\rangle \leftarrow ADD(\langle FP_{global}[j]\rangle ,\langle FP_{S_{i}}[j]\rangle ,PK_{p})$

8:    **end for**

9:   **end for**

10:   **Step 2: Compute Denominator Components**

11:   $a,b,r_{A}^{1},r_{A}^{2}\leftarrow RANDS()$

12:   $\langle TP_{A}\rangle \leftarrow MUL(\langle TP_{global}[last]\rangle ,a)$

13:   $\langle FP_{A}\rangle \leftarrow MUL(\langle FP_{global}[last]\rangle ,b)$

14:   $\langle D_{1}\rangle \leftarrow ADD(\langle TP_{A}\rangle ,r_{A}^{1})$

15:   $\langle D_{2}\rangle \leftarrow ADD(\langle FP_{A}\rangle ,r_{A}^{2})$

16:   $\langle D_{3}\rangle \leftarrow ADD(ADD(MUL(\langle TP_{A}\rangle ,r_{A}^{2})\;+\;MUL(\langle FP_{A}\rangle ,r_{A}^{1})),r_{A}^{1}\;\cdot \;r_{A}^{2})$

17:   **Step 3: Compute Numerator Components**

18:   **for** *j* = 1 to len(D) **do**

19:    $\langle TP_{final}[j]\rangle \leftarrow ADD(\langle TP_{global}[j]\rangle ,\langle TP_{global}[j-1]\rangle ))$

20:    $\langle FP_{final}[j]\rangle \leftarrow ADD(\langle FP_{global}[j]\rangle ,MUL(\langle FP_{global}[j-1]\rangle ,-1))$

21:   **end for**

22:   $\langle N_{3}\rangle \leftarrow 0$

23:   **for** *i* = 0 to len(D) **do**

24:    $r_{i}^{1},r_{i}^{2}\leftarrow RANDS()$

25:    $\langle sTP[i]\rangle \leftarrow MUL(ADD(\langle TP_{final}[i]\rangle ,\langle TP_{final}[i-1]\rangle ),a)$

26:    $\langle dFP[i]\rangle \leftarrow MUL(SUB(\langle FP_{final}[i]\rangle ,\langle FP_{final}[i-1]\rangle ),b)$

27:    $\langle N[i{]}_{1}\rangle \leftarrow ADD(\langle sTP[i]\rangle ,r_{i}^{1})$

28:    $\langle N[i{]}_{2}\rangle \leftarrow ADD(\langle dFP[i]\rangle ,r_{i}^{2})$

29:    $\langle N[i{]}_{3}\rangle \leftarrow ADD(ADD(MUL(\langle sTP[i]\rangle ,r_{i}^{2})\;+\;MUL(\langle dFP[i]\rangle ,r_{i}^{1})),r_{i}^{1}\;\cdot \;r_{i}^{2}\;+\;z_{i})$

30:    $\langle N_{3}\rangle \leftarrow ADD(\langle N_{3}\rangle ,\langle N[i{]}_{3}\rangle )$

31:   **end for**

32:   **Step 4: Partially Decrypt Components**

33:   $\left(\langle N[i{]}_{1}\rangle ,\langle N[i{]}_{2}\rangle )\leftarrow DEC\_P((\langle N[i{]}_{1}\rangle ,\langle N[i{]}_{2}\rangle ),SK_{P_{0}}\right)$

i∈{1,2,…,len(D)}

34:   $\left(\langle D_{1}\rangle ,\langle D_{2}\rangle ,\langle D_{3}\rangle ,\langle N_{3}\rangle )\leftarrow DEC\_P((\langle D_{1}\rangle ,\langle D_{2}\rangle ,\langle D_{3}\rangle ,\langle N_{3}\rangle ),SK_{P_{0}}\right)$

35: **end procedure**

Next, the proxy calculates the final TP and FP values. For each decision point *j* (from the second point onward), the proxy adds the cumulative values from the previous decision point to the current point for $\langle TP_{final}[j]\rangle$. For $\langle FP_{final}[j]\rangle$, the proxy computes the difference between the current and previous FP values. These calculations are performed securely using Paillier operations to preserve data privacy.

From this point on, Algorithm 4 follows the same steps as Algorithm 2 for computing the numerator and denominator components, as well as partially decrypting these components. Specifically, the proxy computes randomized encoding components for the numerator and denominator using securely scaled true positive and false positive values. The proxy then partially decrypts these randomized components using its partial private key $SK_{S_{P}}$ and shares them back with the stations.

#### Final computation.

DPPA-AUC computation utilizes the same Eq 5 as the DPPE-AUC to compute the final AUC value locally at each station.

### 3.4 Security assumptions and guarantees

Our protocol’s security relies on specific assumptions about the behavior of the participating entities and the cryptographic primitives employed. This section outlines our threat model, the roles of each party, and the guarantees provided.

#### Party roles and trust model.

The protocol involves three types of entities: Data Stations (*S*_*i*_), an Initialization Station (*S*_0_), and a Proxy Station (*S*_*P*_). We operate under a **semi-honest** (or honest-but-curious) threat model for all parties. This assumption means that all stations correctly follow the protocol’s steps but may attempt to infer additional information from the messages they receive during the computation.

- **Data Stations ($S_{1},...,S_{n}$) and Initialization Station (*S***_**0**_): These are assumed to be semi-honest. They hold private data (prediction scores and true labels) and are trusted to follow the protocol, but they might try to learn about the data at other stations.
- **Proxy Station (*S***_***Proxy***_): The proxy is also assumed to be a semi-honest party. It facilitates the secure aggregation but does not contribute its own data.

#### Collusion assumptions.

A critical assumption in our security model is that there is **no collusion between the proxy station (*S***_***Proxy***_) **and any of the data stations (*S***_***i***_). An adversary controlling only the proxy or one or more data stations (but not both) cannot compromise the privacy of other honest stations’ raw data.

#### Security guarantees and information leakage.

Under the semi-honest and no-collusion model, our protocol provides strong cryptographic guarantees for the confidentiality of sensitive inputs. The security of our scheme depends on the security properties of the modified Paillier system and randomized encoding. The Paillier cryptosystem provides both one-wayness and semantic security [11], while the security proofs for the randomized encoding are provided by [17].

- **Protected Information:** The true labels and raw prediction scores from each data station remain private. Labels and flags are encrypted with a modified Paillier cryptosystem, and since each station only holds a share of the private key, no single station (including the proxy) can decrypt them.
- **Information Leakage:** The proxy station can observe the masked prediction values. However, these values are obscured using randomization and the injection of dummy samples, which prevents the proxy from inferring the true prediction scores or their distribution. The proxy also handles encrypted true positive (TP) and false positive (FP) values but cannot decrypt them to learn the underlying counts.
- **Collusion Vulnerability:** If an adversary compromises both the proxy station and at least one data station, they could combine the partial private keys to decrypt all labels, flags, and predictions across the entire network.

This security analysis applies to both DPPE-AUC and DPPA-AUC, since the only methodological difference (exact vs. approximate thresholds) does not affect the underlying cryptographic primitives or trust assumptions.

#### Limitations and mitigation strategies.

Protecting against collusion between the proxy and a data station is a limitation of the current security model and is considered out of scope for this work. However, this vulnerability could be mitigated in future work by decentralizing the proxy’s trust and functions. Potential enhancements include:

- **Threshold Cryptography:** Integrating a threshold version of the Paillier cryptosystem would distribute the decryption key among multiple parties, requiring a certain threshold of them to collaborate for decryption and thus removing the single point of trust at the proxy.
- **Secure Multi-Party Computation (MPC):** The proxy’s operations, such as sorting and aggregation, could be redesigned using MPC protocols. This would allow these computations to be performed jointly by multiple parties without any single entity having access to intermediate data, thereby eliminating the need for a trusted proxy.

### 3.5 Computational complexity summary

To clarify the scalability characteristics of our protocols, we summarize the asymptotic runtime complexity of DPPE-AUC and DPPA-AUC in Table 2. The main difference lies in how the number of thresholds is determined. For DPPE-AUC, thresholds are derived directly from the prediction values, including possible tie conditions. This results in a runtime that grows linearly with both the number of samples and the number of participants. In contrast, DPPA-AUC uses a fixed, user-defined number of thresholds (decision points), leading to runtime that is constant with respect to the sample size but still increases linearly with the number of participants. This behaviour highlights the trade-off between exactness and efficiency that we further evaluate in the experiments.

**Table 2:** Asymptotic runtime complexity of DPPE-AUC and DPPA-AUC in terms of number of samples (*n*), number of participants (*s*), and number of thresholds (*K*).

Method	Samples (*n*)	Participants (*s*)	Thresholds (*K*)
DPPE-AUC	O(n·s)	Linear	Data-derived thresholds
DPPA-AUC	O(s·K)	Linear	Fixed, user-defined

### 3.6 Showcase and experiments

To evaluate the efficacy and privacy-preserving capabilities of our DPPE- and DPPA-AUC methods, we conducted experiments using both real-world HIV-1 V3-loop sequence data and synthetic datasets, benchmarking against ground truth (GT) AUC using the *roc_auc_score* function from the scikit-learn metrics library [26]. We performed all experiments and showcases on a local computer (Apple Mac mini, M4Pro, 48GB memory).

#### Showcase: HIV-1 coreceptor binding prediction.

**Fig 2:**
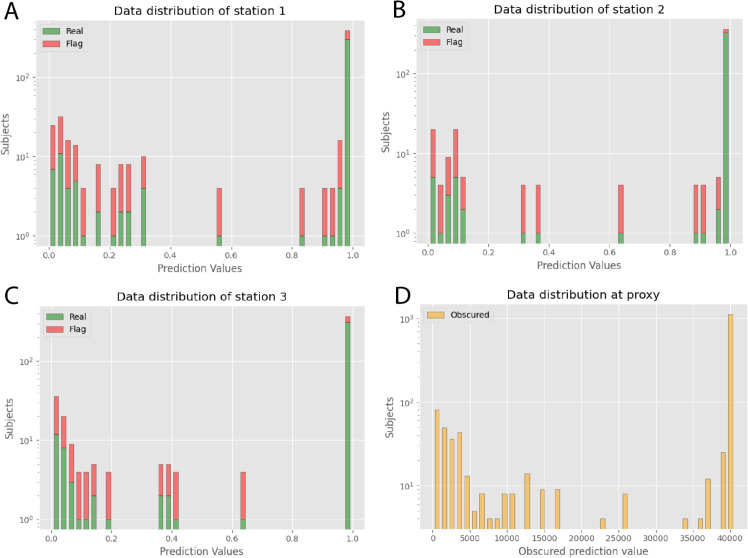
(A–C) Input data site 1-3. Prediction value distribution at Station 1 to 3, highlighting the balance between ‘Real’ and ‘Flag’ categories. Peaks at both ends of the prediction range indicate high variability in the dataset. **(D) Input data at Proxy**: Obscured prediction value distribution at the proxy station, showing transformed prediction data aggregated from all sites. The distribution indicates effective obfuscation of individual prediction values.

For the primary showcase, we utilized 10462 HIV-1 V3-loop sequences annotated with coreceptor binding data [27]. The binary classification task is to predict binding preferences for coreceptors *CCR*5 and *CXCR*4, with data distributed across three stations for decentralized analysis Fig 2. Each station trained a local model using its partitioned dataset, while a 10% holdout set was maintained for independent evaluation. This setup allowed us to test our method’s effectiveness in preserving data privacy across distributed sites.

For performance reasons, we employed a flag patient generation approach, injecting synthetic samples only within the value range of actual patient data to not increase the number of thresholds. Accuracy is not affected by the generated dummy data, since they all have labels and flag values of zero before encryption.

We computed the corresponding DPPE- and DPPA-AUC components for our method and derived the difference to the GT. Our experiments included the DPPE- and DPPA-AUC method compared in terms of time and deviations from GT AUC score. The results in Table 3 highlight a trade-off between accuracy and computational efficiency: DPPE-AUC achieves near-perfect accuracy with a minimal difference from the GT but incurs a higher computational cost, while DPPA-AUC demonstrates faster runtime with a slightly larger but acceptable deviation from the GT AUC.

**Table 3:** Comparison of DPPE-AUC and DPPA-AUC methods based on runtime, the difference from the ground truth AUC, and the number of thresholds used.

Method	Time (s)	Deviation to GT	Thresholds
DPPE-AUC	38.71	1.11E-16	145
DPPA-AUC	26.499	0.0037	100

For each run, Across ten runs, the average difference between the GT and DPPE-AUC values was 10^−16^, likely attributable to floating-point arithmetic precision limits in Python [28].

#### Experiments.

While the showcase demonstrated the applicability of our approach in a realistic federated setting, it also revealed limitations inherent to real-world data—specifically, the dominance of high AUC scores and repetitive prediction values. These characteristics introduced bias, making conducting a meaningful performance evaluation across varying sample sizes and station counts difficult.

To systematically investigate our methods under controlled conditions, we conducted a series of synthetic experiments designed to more accurately examine the impact of these values. At each station, subjects were randomly assigned prediction values between 0 and 1 and labels from the set 0,1. Random flag patients were created identically to those in the showcase. We compared the GT AUC with the DPPE- and DPPA-AUC, excluding flag samples in the GT calculation to validate our method. Across experiments, differences between GT and DPPE-AUC values remained consistently at 10^−18^. Precisely in experiment 1 $-3.1720^{-18}\;\pm \;6.0430^{-17}$ (mean ± std) over all 70 runs, and for experiment 2 $-1.22002^{-17}\;\pm \;4.3632^{-17}$ over all 90 runs.

#### Experiment 1.

In this experiment, we analyzed the runtime performance of both methods compared to GT AUC. The total dataset size was fixed at 1500 subjects, combining both actual and dummy (flag) patients. The experiment aimed to simulate a realistic PHT-meDIC environment, where an increasing number of participating stations (or input parties) operate decentralized. As the number of sites increased, the sample size per site was proportionally reduced per site.

This configuration allowed us to analyze how computational efficiency scales with the number of sites while maintaining a constant total sample size. Each site independently computed its local AUC contribution iteratively, with the overall execution time determined by the combined decentralized encryption procedures. The synthetic data generation in this experiment mimicked a real-world scenario by introducing biases around prediction values near 0 and 1. Flag data was generated only for existing subjects to obscure the data distribution, ensuring no increase in the number of threshold values. This approach effectively minimized computational overhead while preserving data realism.

**Fig 3:**
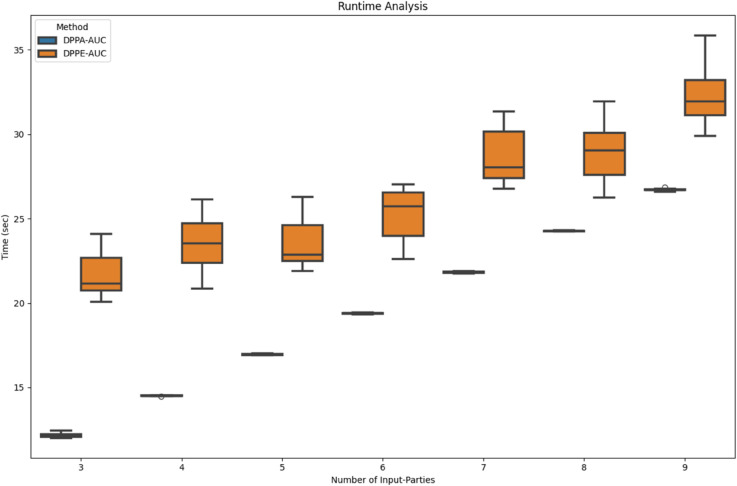
Increasing number of input parties. Runtime performance over 10 runs of DPPE-AUC and DPPA-AUC methods with a fixed total dataset size of 1500 subjects. The number of participating sites (input parties) varies between 3 and 9, illustrating the scaling behavior of both methods. While execution time increased with more stations, both methods maintained robust performance.

As expected, the runtime increased with the number of participating sites in Fig 3 due to decentralized computations overhead. Both methods demonstrated robust performance, though DPPE-AUC required slightly more time than DPPA-AUC due to its complete processing of input data, while DPPA-AUC only uses 100 approximation points.

#### Experiment 2.

In this experiment, we examined how runtime performance scales with increasing sample sizes while keeping the number of stations fixed at three. This setup evaluated the computational scalability of the DPPE-AUC method compared to the DPPA-AUC method as the amount of data per station increases.

**Fig 4:**
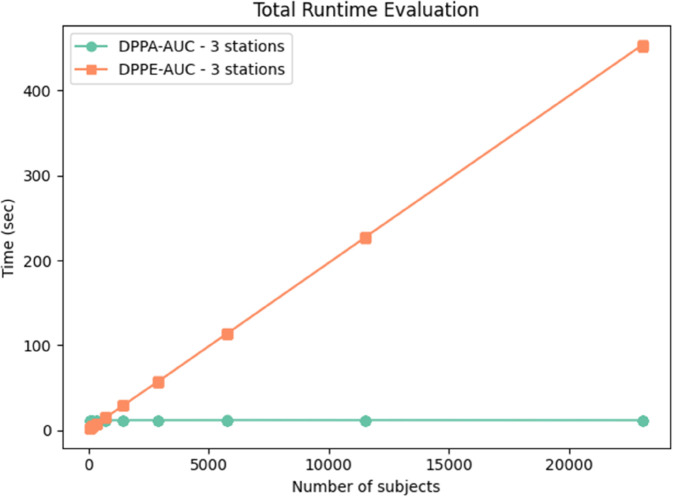
Increasing number of input samples. Runtime evaluation of DPPE-AUC and DPPA-AUC methods with a fixed number of stations (3) and increasing dataset sizes (up to 23,000 subjects). DPPE-AUC exhibits linear scaling with increasing sample sizes, while DPPA-AUC maintains constant runtime with respect to samples due to its reliance on a fixed number of decision points.

The evaluation considered up to 23,000 subjects, reflecting a practical sample size in typical PHT-meDIC studies. The results in Fig 4 demonstrated a linear increase in execution time for the DPPE-AUC method as the sample size grew. This behavior aligns with the computational demands of DPPE-AUC, where encryption and processing steps scale directly with the input data to process. In contrast, DPPA-AUC maintains constant runtime with respect to the number of samples, since its computation depends only on a fixed number of decision thresholds. However, its overall runtime still grows with the number of participating stations (Fig 4). The data generation followed a uniform random distribution, representing a worst-case scenario in terms of computational overhead.

These findings highlight distinct scalability characteristics for the two methods. While DPPE-AUC is well-suited for moderate-scale datasets, DPPA-AUC’s fixed runtime offers a compelling advantage for large-scale applications where efficiency and predictable computational costs are critical.

## 4 Discussion

The primary challenge identified in the current implementation for both methods is the increase in runtime as the number of participants grows, largely due to the iterative execution within the PHT-meDIC framework. To mitigate this, transitioning to a federated execution of the station-side algorithms and final computation steps. This would enable parallel processing, thereby significantly reducing the overall total runtime, as evidenced by our experimental results. Another issue related to the robustness of the sequential execution of PHT-meDIC is that if any station is missing, subsequent stations cannot proceed with the analysis, which the user must resubmit.

Furthermore, the runtime performance of DPPE-AUC strongly depends on how prediction values are distributed. If every prediction value is unique, the sorting and processing steps reach their highest overhead. To both obscure the real distribution and limit uniqueness, our current approach inserts “dummy” prediction values drawn from the existing set, thereby reducing the overall number of distinct values. While this technique conceals the original distribution effectively, it remains a straightforward implementation. In general, the choice of dummy data does not affect the accuracy but only the runtime of the method. Consequently, there is still significant room for optimization, particularly at the proxy station during final sorting and processing, to further enhance execution speed. However, in scenarios where millions of samples need to be analyzed, the DPPA-AUC approach is always recommended due to significant performance overheads.

The potential of achieving marginal AUC improvements, such as the ≈0.0037 difference provided by DPPE-AUC compared to the DPPA-AUC method, is mainly relevant in critical healthcare domains. In rare disease diagnoses, small gains in AUC can significantly improve early detection rates and reduce misdiagnoses [29]. Similarly, such improvements enhance early tumor detection in radiology and lower false negatives [30]. In ICU sepsis prediction, even minor performance increases can directly impact timely interventions and patient outcomes [31]. Furthermore, in personalized medicine and adverse drug reaction prediction, higher precision minimizes unnecessary treatments and associated risks [32, 33]. These examples highlight the importance of fine-grained performance gains in machine learning models for healthcare applications, emphasizing the value of DPPE-AUC.

## 5 Conclusion

In this work, we introduced DPPE-AUC and DPPA-AUC, two cryptographic protocols that enable the computation of the global AUC in distributed and privacy-sensitive settings. DPPE-AUC calculates the AUC exactly, making it particularly useful when high-precision model assessment is important; it maintains linear scaling with the dataset size. DPPA-AUC instead relies on a sampling strategy with a fixed set of thresholds, which yields constant runtime with respect to the number of samples. Its overall runtime, however, still depends on the number of participating sites. This design makes DPPA-AUC especially suitable for larger or more diverse datasets where computational efficiency and predictable cost are critical. Both methods are fully integrated into the PHT-meDIC framework, highlighting their practical feasibility. The methods are agnostic to the data modality as long as the task can be framed as a binary classification with prediction values in ℝ, labels in [0,1] and no ties pre-bucketed.

By combining modified Paillier encryption, symmetric and asymmetric cryptography, and randomized encoding techniques, these protocols protect sensitive model predictions and labels while still allowing global performance metrics to be derived. This leads to a robust and flexible system that meets the privacy demands of multi-institutional collaborations in healthcare and beyond. In future work, we plan to optimize the protocols further by employing parallelization strategies, which could substantially reduce runtime in large-scale distributed networks. Through such optimizations, we aim to make secure, privacy-preserving performance evaluation an even more powerful tool for real-world medical and scientific applications. Another direction for future research is to enhance our protocols against scenarios where the proxy station might collude with a participating data station, a situation currently outside our security model. Strengthening the system against such collusion would further bolster its security guarantees. Potential avenues to explore include:

- Investigating the integration of threshold cryptography to distribute trust.
- Redesigning critical proxy operations using Secure Multi-Party Computation (MPC).
- Exploring alternative advanced cryptographic primitives or architectural modifications within the PHT-meDIC framework.

Addressing these aspects will be crucial for deploying privacy-preserving AUC computations in environments requiring resistance to more complex adversarial models. We also plan to address security and runtime optimization concerns within another federated learning platform, FLAME, as part of the MII PrivateAIM project. The main optimization in FLAME are achieved by parallel computation at all sites and by integrating the Proxy station functionality in the underlying FLAME-SDK, such the initialization steps can be skipped.
